# Isolation and evaluation of multi-functional properties of lactic acid bacteria strains derived from canine milk

**DOI:** 10.3389/fvets.2024.1505854

**Published:** 2024-11-28

**Authors:** Yunjiang Liu, Yueyan Zeng, Li Chen, Jialiang Xin, Zhijun Zhong, Haifeng Liu, Hualin Fu, Ziyao Zhou, Guangneng Peng

**Affiliations:** ^1^Key Laboratory of Animal Disease and Human Health of Sichuan Province, College of Veterinary Medicine, Sichuan Agricultural University, Chengdu, China; ^2^Key Laboratory of Qinghai-Tibetan Plateau Animal Genetic Resource Reservation and Utilization, Ministry of Education, Southwest Minzu University, Chengdu, Sichuan, China

**Keywords:** lactic acid bacteria, canine milk, adhesion, antioxidant capacity, metabolite

## Abstract

**Introduction:**

Lactic acid bacteria (LAB) are Gram-positive bacteria that produce lactic acid during fermentation, with some strains enhancing host health by modulating the gut microbiota, boosting immune responses, and reducing inflammation.

**Methods:**

In this study, 6 LAB strains were isolated from two dog milk samples, and their probiotic properties were comprehensively evaluated. The evaluation included growth properties, stress resistance, antipathogen activity, adhesion activity, safety assessment, antioxidant capacity, and prebiotic metabolites assessment.

**Results:**

In comparison to the control strain *Lactobacillus rhamnosus* LGG, all 6 LAB isolates exhibited favorable probiotic properties. Additionally, the results of the antioxidant tests indicated that these strains demonstrated high tolerance to 0.5 mmol/L H_2_O_2_ and exhibited significant scavenging abilities for the free radicals 1,1-diphenyl-2-trinitrophenylhydrazine (DPPH) and hydroxyl (OH^−^). Furthermore, the 6 LAB isolates were found to produce elevated concentrations of prebiotic metabolites, including exopolysaccharides (EPS), γ-aminobutyric acid (GABA), and bile salt hydrolase (BSH).

**Discussion:**

This study presents a comprehensive analysis of LAB isolates derived from canine milk. These isolates exhibited multifunctional properties, with strain L221 performing the best overall, making it a promising candidate for probiotic use in dogs.

## Introduction

The definition of probiotics, according to the World Health Organization, is that they are live microorganisms that provide health benefits to the host when administered in sufficient quantities (1). Lactic acid bacteria (LAB), including *Lactobacillus, Lactococcus, Pediococcus, Enterococcus*, and *Streptococcus*, are commonly found in milk, feces, fermented dairy products, and the gastrointestinal tract, with certain strains exhibiting probiotic potential (2–6). Milk and its products are generally considered the main source of LAB and have always been widely used to isolate probiotics (7, 8). Numerous studies have shown that certain LAB can improve digestion, maintain intestinal microbiota balance, preventing infections, and boost the immune system (3–6, 9, 10). Since the discovery of the multifunctional properties of certain LAB strains, the incorporation of LAB into food, cosmetics, and medicine has become increasingly popular (11–16).

Dog is a popular choice for household pets worldwide. Changes in diet, environment, and weakened immune systems can make them vulnerable to digestive disorders, such as gastroenteritis, pancreatitis, and inflammatory bowel disease. These conditions can disrupt the body's intestinal microbiota, leading to symptoms such as vomiting, diarrhea, allergies, and obesity (17, 18). As the number of pet dogs increases and human life quality improves, pet owners are increasingly considering scientific feeding methods and functional pet products to meet their dogs' health and nutritional needs (19). LAB is commonly used as a food additive or nutritional supplement by pet owners to improve the health of dogs due to its beneficial functions (10).

Furthermore, while existing studies have primarily focused on the basic properties of LAB strains, such as their stress resistance, antibacterial activity, and adhesion capabilities, there remains a significant gap in the evaluation of their antioxidant properties and production of beneficial metabolites, particularly in the context of canine health (20–23). The current research aims to bridge this gap by not only screening for LAB strains with multifunctional potential derived from canine milk but also by extensively assessing their antioxidant capacity and the production of probiotic metabolites, which are crucial for their therapeutic efficacy. By focusing on these underexplored properties, this study aims to identify LAB strains that possess enhanced safety profiles and broader probiotic properties, tailored specifically for canine health.

## Materials and methods

### Sample collection

Milk samples, ~1 mL each, were obtained from two healthy domestic Beagle dogs that had not been administered antibiotics or probiotics for at least 1 month prior to sample collection. The samples were immediately transported to the laboratory under cold chain conditions and stored at 4°C until further analysis.

### Isolation and purification and species identification

The Isolation, Purification and Species Identification was conducted based on Zhang's report with minor adjustments (24). To isolate LAB strains, 0.1 mL of canine milk was inoculated onto de Man, Rogosa, and Sharpe (MRS) agar plates (Hopebio, Qingdao, China) and incubated aerobically at 37°C for 24–72 h until distinct colonies appeared. Isolated colonies were subjected to three rounds of purification by re-culturing on MRS agar plates, followed by enrichment in MRS broth. All subsequent bacterial counting work is completed by Halo counter HD-4 Cell Counter (Hiscore Inc., China). The 16S rRNA genes of all isolates were amplified using universal primers 27F (5′-AGGTTTTTGATCCTGGCCAG-3′) and 1492R (5′-TACGACTTAACCCCAATCGC-3′). The resulting amplicons were sequenced by Sangon Bioengineering (Chengdu) Co., Ltd., and the sequences were aligned using BLAST against GenBank bacterial nucleic acid databases. Species identification was performed using MEGA6 software (Mega Limited, Auckland, New Zealand), employing the neighbor-joining method and the Kimura 2-parameter model to construct a phylogenetic tree. A sequence similarity threshold of >99% was used to define species.

### Growth performance evaluation

Growth performance was evaluated by measuring the growth curve of LAB isolates as previously described by Liu et al. (25). A 50 μL aliquot (1%) of LAB culture in the mid-exponential phase was inoculated into 50 mL of fresh MRS broth and incubated at 37°C for 48 h. Optical density (OD600) measurements were taken every 2 h during the first 12 h and subsequently every 4 h up to 48 h.

### Tolerance for simulating the GIT environment

Tolerance of the LAB isolates to simulated gastrointestinal conditions was tested according to the method of Zhang et al. (24), with slight adjustments to simulate the specific gastrointestinal fluid conditions in dogs. The LAB isolates were incubated in MRS broth for 12 h. Then, 10 ml of the fresh cultures were centrifuged at 8,000 × g for 10 min at 25°C to obtain bacterial cells. Bacterial cells were suspended in 10 mL of simulated gastric juice and incubated at 37°C for 3 h. Cells were then harvested by centrifugation and transferred into 10 mL of simulated intestinal juice for incubation at 37°C for an additional 4 h. Bacterial survival at 0, 3, and 7 h was assessed using colony-forming units (CFU) determined by plate counting. Survival rates were calculated as the ratio of surviving CFUs at each time point to the previous time point count.

## Antipathogenic activity detection

### Antibacterial activity

The Antibacterial activity detection was conducted based on Zhang's report with minor adjustments (24). The antibacterial potential of LAB isolates was tested using the Oxford cup method against four common enteropathogens: *Staphylococcus aureus* ATCC 25923, *Escherichia coli* ATCC 25922, *Salmonella enterica* H9812, and *Pseudomonas aeruginosa* PAO1. Cell-free supernatants (CFS) were obtained by centrifuging LAB cultures at 4,500 × g for 10 min and filtering the supernatant through a 0.22 μm sterile filter. The pathogenic bacteria were cultured in LB broth, diluted to about 10^7^ CFU/mL, and 100 μL of each suspension was spread onto LB agar plates. Three sterile Oxford cups were placed on each plate and filled with 200 μL of CFS. Plates were incubated at 37°C for 24 h, and inhibition zones were measured using a caliper to determine antibacterial activity.

### Co-aggregative ability with pathogens

Co-aggregation of LAB with the four pathogens was assessed by mixing equal volumes (2 mL) of LAB and pathogen suspensions, followed by incubation at 37°C for 2 h. Absorbance (A_600_) was measured before and after incubation for both individual and mixed cultures. The co-aggregation rate was calculated using the formula: Co-aggregation rate (%) = 1 – A_mix_ / [ (A_LAB_ + A_pathogen_) / 2] × 100.

## Adhesion activity detection

### Auto-aggregation activity

According to the report by Zhang et al. (24). Auto-aggregation activity was measured by adjusting the LAB culture to 1 × 10^8^ CFU/mL in phosphate-buffered saline (PBS). After incubation at 37°C for 6 h, the OD600 of the upper layer of the bacterial suspension was measured, and auto-aggregation was calculated as: Auto-aggregation rate (%) = 1 – (A_1_/A_0_) × 100, where A_0_ is the initial OD and A_1_ is the OD at 6 h.

### Cell surface hydrophobicity

The hydrophobicity of LAB strains in various organic solvents was determined using a modified version of the method reported by Kos (26). The LAB strains that were activated were introduced into MRS liquid medium at a concentration of 1% (v/v) and incubated overnight. Afterward, they were centrifuged at 8,000 × g for 10 min at 4°C to collect the microorganisms. The microorganisms were then washed three times with sterile phosphate-buffered saline (PBS, pH = 7.4) and resuspended in PBS. The absorbance of the lactobacilli suspension was adjusted to OD600 = 0.60 ± 0.05 (A_0_). LAB strains suspension (3 mL) was mixed with 1 mL of different organic solvents (ethyl acetate, xylol, and trichloromethane), vortexed and shaken for 2 min, and then allowed to stand for 20 min. The OD value of the aqueous phase was measured at 600 nm using a UV spectrophotometer (A_1_). The experiment was repeated three times. The hydrophobicity of LAB was calculated using the following formula: Hydrophobicity rate (%) = (1-A_1_/A_0_) × 100.

### Adhesion to Caco-2 cells

The Adhesion to Caco-2 cells was conducted based on Wang's report with minor adjustments (27). Caco-2 cells were cultured in a flask until they reached a sub-confluent state of 80–90%. They were then digested with 0.25% trypsin and counted using a hemocytometer. The concentration of viable cells was adjusted to 1 × 10^5^ cells/mL (V_C)_ using DMEM medium. The cell suspension was added to a 12-well cell culture plate at a volume of 1 mL per well. The plate was then incubated at 37°C and 5% CO_2_ until the cells formed a confluent monolayer, typically within 24–48 h. Prior to the adhesion assay, the cells were cultured for 1 day, and the medium was replaced with high-glucose DMEM (without antibiotics) to avoid any influence of antibiotic resistance on the adhesion assay. At the beginning of the assay, the cells were washed three times with sterile PBS and 1 mL of a LAB suspension at a concentration of 1 × 10^8^ CFU/mL (V_0_) was added to each well. The cells were then incubated at 37°C and 5% CO_2_ in a constant-temperature cell culture incubator for 2 h. After the incubation period, the cells were washed three times with sterile PBS to remove any unattached LAB. The cells were digested using 0.25% trypsin, which should be handled with care due to its potential hazards. After complete digestion, the cells were collected and subjected to 10-fold gradient dilution. The number of viable adherent LAB after dilution on MRS solid media was determined using plate colony counting (V_1_) after incubating the plates at 37°C for 48 h in an anaerobic jar. The experiment was conducted three times. The adhesion rate and adhesion index of LAB to Caco-2 cells were calculated using the following formula: Adhesion rate (%) = (V_1_ / V_0_) × 100; Adhesion index (CFU/cell) = V_1_ / V_C_.

### Determination of biofilm forming ability

Research indicates that LAB with a strong ability to form biofilm exhibit better resistance to heat and freezing (28). The biofilm-forming ability of LAB strains was determined using crystal violet staining (29). The concentration of LAB was adjusted to 1 × 10^7^ CFU/mL. The LAB suspension was inoculated into 96-well cell culture plates at a volume of 200 μL per well. The plates were then incubated in a 37°C incubator for 24 h to form a stable biofilm. Blank MRS liquid medium was used as a control. The bacteria were washed three times with sterile PBS to remove the planktonic bacteria, and then dried at room temperature for 15 min. Next, they were fixed in 200 μL of methanol solution for 15 min and dried again at room temperature for 10 min. The sample was immersed in a 1% crystal violet solution (200 μL) for 20 min, rinsed three times with distilled water, and air-dried for 10 min. It was then eluted in a 33% acetic acid solution (200 μL) for 10 min, and the resulting decolorized solution was analyzed at a wavelength of 595 nm using an enzyme marker. The OD value of the decolorized solution at 595 nm was measured using an enzyme counter (the control was recorded as A_0_ and the LAB were recorded as A). The strength of LAB biofilm formation ability was evaluated based on the following criteria: no biofilm formation ability (–): A <A_0_; weak biofilm forming ability (+): A_0_ <A ≤ 2A_0_; moderate biofilm forming ability (++): 2A_0_ <A ≤ 4A_0_; and strong biofilm forming ability (+++): A > 4A_0_. The experiments were repeated three times.

## Safety assessment

### Hemolytic activity

The Safety assessment test was conducted based on Zhang's report with minor adjustments (24). LAB isolates were streaked onto blood agar plates and incubated at 37°C for 48 h. Staphylococcus aureus ATCC 25923 was used as a positive control. Hemolysis was assessed by the presence of clear zones (β-hemolysis), green zones (α-hemolysis), or no zones (γ-hemolysis).

### Antibiotic susceptibility

The susceptibility of the selected LAB strains to antibiotics was assessed using the disc-diffusion test. A total of 14 antimicrobials (Shunyoubio, Shanghai, China) were tested, including penicillin G (P, 10 μg), ampicillin (AMP, 10 μg), amoxicillin (AML, 25 μg), erythromycin (E, 15 μg), Cefuroxim (CXM, 30 μg), and cefotaxime (CTX). Oxacillin (OX, 5 μg), Cefazolin (KZ, 30 μg), Norfloxacin (NOR, 5 μg), Rifampicin (RD, 5 μg), clindamycin (DA, 10 μg), chloramphenicol (C, 30 μg), tetracycline (TE, 30 μg), and vancomycin (VA, 30 μg). Fresh overnight cultures of each LAB strain were diluted to a concentration of 10^8^ CFU/ml. Subsequently, 100 μl of the diluted cultures were spread on MRS agar plates and dried. Three uniform antibiotic discs were manually placed on the surface of the dried MRS plates. The plates were then inverted and incubated for 48 h under anaerobic conditions at 37°C. Antibiotic susceptibility was classified as resistant (R), moderately susceptible (M), or sensitive (S) based on the diameter of the zone of inhibition (mm) according to the parameters of the Clinical and Laboratory Standards Institute (65).

## Antioxidant capacity assessment

Inoculate 2% (v/v) of the activated LAB culture into the MRS liquid medium. After an overnight culture, centrifuge the mixture at 4°C at 8,000 × g for 10 min. Collect the supernatant to obtain a cell-free supernatant. Then, resuspend the pellet in PBS and adjust the LAB concentration to 1 × 10^9^ CFU/mL to obtain a bacterial suspension.

### Tolerance to H_2_O_2_

The tolerance of LAB to H_2_O_2_ was measured using the method reported by Xiong et al. (30). A liquid culture of LAB with a concentration of 1 × 10^8^ CFU/mL was inoculated into MRS liquid culture medium containing 0, 0.5, 1.0, 1.5, and 2.0 mmol/L H_2_O_2_ at an inoculum volume of 2% (v/v). The mixture was incubated for 8 h at 37°C in a constant-temperature incubator, and the OD value of the culture medium was measured at a wavelength of 600 nm using a UV spectrophotometer. The experiment was repeated three times.

### DPPH radical scavenging ability

The ability of LAB to scavenge the 1,1-diphenyl-2-picrylhydrazyl (DPPH) free radical was determined using the method described in the literature (31). Two milliliter of 0.2 mmol/L DPPH absolute ethanol solution was added to a centrifuge tube containing 1 mL of cell-free supernatant or bacterial suspension of lactic acid bacteria. The mixture was vortexed and left to react for 30 min at room temperature in the dark at 4°C. The supernatant was collected by centrifuging at 8,000 × g for 10 min. The OD value of the supernatant was measured at a wavelength of 517 nm using a UV spectrophotometer (OD_sample_). Anhydrous ethanol was used as the blank group instead of DPPH absolute ethanol solution (OD_blank_). Distilled water was used as the control group instead of the reaction sample (OD_control_). The experiment was conducted three times. Afterward, we calculated the DPPH free radical scavenging rate of LAB using the following formula: DPPH free radical scavenging rate (%) = [1– (OD_sample_ – OD_blank_) / OD_control_] × 100.

### Determination of OH^−^ free radical scavenging ability

The OH^−^ scavenging capability was determined following the protocol outlined by Alam et al. with certain adjustments (32). Five hundred microliters of LAB cell-free supernatant or suspension were added to a centrifuge tube, followed by 1 mL of 0.1% 1,10-phenanthroline, 1 mL of PBS, 1 mL of 2.5 mmol/L FeSO4, and 1 mL of 20 mmol/L H_2_O_2_. After incubating for 1.5 h in a water bath set at 37°C, we measured the OD536 of the reaction mixture (OD_sample_). In the blank group, we substituted a consistent volume of absolute ethanol for H_2_O_2_ (OD_blank_). Similarly, in the control group, we replaced the sample solution with an equivalent volume of distilled water (OD_control_). We determined the OH^−^ radical scavenging rate using the following formula: OH^−^ free radical scavenging rate (%) = [(OD_sample_ − OD_control_)/ (OD_blank_− OD_control_)] × 100.

### Determination of O^2−^ free radical scavenging ability

The ability of LAB to scavenge O^2−^ free radicals was evaluated using the method described by Liu et al. (33). LAB cell-free supernatant or bacterial suspension (100 μL) was mixed with 2.8 mL of 0.05 mol/L Tris-HCl (pH 8.2) and 100 μL of 0.05 mol/L pyrogallol. The mixture was vortexed and incubated at 25°C in the dark. After 4 min, the reaction was stopped by adding 1 mL of 8 mol/L HCl. To measure the OD value of the reaction solution at a wavelength of 320 nm (OD_sample_), use a UV spectrophotometer. Adjust to zero with distilled water, which replaces the sample for reaction as a control group (OD_control_). The experiment was repeated three times. Then, calculate the O^2−^ free radical scavenging rate of LAB using the following formula: O^2−^ free radical scavenging rate (%) = [1 − OD_sample_ / OD_control_] × 100.

## Metabolite determination

### Determination of exopolysaccharides production capacity

The ability of LAB to produce EPS was determined using the method described by Ren et al. (34). To prepare the cell-free LAB supernatant, follow the same procedure as in the antioxidant test, as previously described. Mix the supernatant with 800 mg/mL trichloroacetic acid to obtain a final concentration of 40 mg/mL. Incubate the mixture at 4°C in a temperature-controlled incubator overnight. Centrifuge the mixture at 4°C at 8,000 × g for 10 min to collect the supernatant. Add 250 μL of 6% phenol solution and 1 mL of concentrated sulfuric acid to 250 μL of supernatant. Mix well in an ice-water bath and cool to room temperature. Next, add 200 μL of the reaction solution to a 96-well cell culture plate and measure the OD value of the reaction solution at a wavelength of 490 nm using a microplate reader, with the blank set to the reagent mixture without the supernatant. To measure the concentration of EPS produced by LAB, prepare a standard curve using glucose solutions with concentrations of 3.125, 6.25, 12.5, 25, 50, and 100 mg/L in the presence of trichloroacetic acid, phenol, and sulfuric acid. Repeat the experiment three times to ensure accuracy.

### Determination of gamma-aminobutyric acid production ability

The ability of LAB to produce GABA was determined using the Berthelot colorimetric method as reported by Zhang et al. (35). The LAB strains were activated and then introduced into a fermentation medium containing glucose, yeast extract, and peptone (GYP), with an inoculation volume of 2% (v/v). After being cultured overnight, the LAB were centrifuged at 8,000 × g for 10 min at 4°C. The supernatant should be collected and mixed with 200 μL of 0.2 mol/L borate buffer (pH 9.0), 1 mL of 6% phenol, and 0.4 mL of sodium hypochlorite solution with an available chlorine content of 5.5%. After adding these components, vortex the mixture. Place the mixture in a boiling water bath for 10 min, followed by an ice water bath for 20 min. Then, add 2 mL of a 60% ethanol solution and mix thoroughly using a vortex. Measure the OD value of the reaction solution at a wavelength of 645 nm using a UV spectrophotometer and adjust to zero with distilled water. To calculate the concentration of GABA produced by LAB, draw a standard curve using GABA standards with concentrations of 0, 0.2, 0.4, 0.6, 0.8, and 1.0 g/L. Repeat the experiment three times.

### Determination of bile salt hydrolase producing ability

The production of BSH by LAB was determined following the method described in Wang et al. (36). The resulting supernatant was collected to obtain a cell-free extract. To prepare the antioxidant test samples, the bacterial suspension was mixed with 10 mmol/L dithiothreitol, sonicated for 10 min in an ice bath, and then centrifuged at 4°C and 8,000 × g for 10 min. To 10 μL of LAB cell-free supernatant or cell-free extract, add 180 μL of 0.1 mol/L PBS (pH 6.0) and 10 μL of 0.1 mol/L sodium taurocholate solution. Then, add 200 μL of 15% trichloroacetic acid and react for 1 min. Centrifuge the mixture at 4°C, 8,000 × g for 10 min and collect the supernatant. Add 100 μL of the supernatant to 1.9 mL of ninhydrin chromogenic solution and vortex. Vortex the mixture and place it in a 37°C water bath for 30 min. Put the mixture in a boiling water bath for 15 min and then in an ice water bath for 3 min. The OD value of the reaction solution was measured at a wavelength of 570 nm using a UV spectrophotometer. Trichloroacetic acid was added to the sample, followed by the addition of sodium taurocholate solution as a control group for the reaction. A standard curve was constructed using glycine standards at concentrations of 0, 0.1, 0.2, 0.3, 0.4, and 0.5 μmol/L. Enzyme activity was defined as the production of 1 μg of glycine per minute at 37°C, with 1 unit of enzyme activity being equivalent to this amount. The experiment was repeated three times.

## Results

In this study, six LAB strains were isolated from the milk of two dogs. These isolates were phenotypically characterized and the results are summarized in Supplementary Table 1. These strains were identified as Gram-positive, rod-shaped or Cocci-shaped, and catalase-negative bacteria. To identify potential probiotic LAB candidates, using the well-characterized reference strain *Lactobacillus rhamnosus* (LGG) as a control, the following assessments were performed on these six strains: species identification, growth performance, stress resistance, antipathogenic activity, adhesion activity, safety assessment, antioxidant capacity, and metabolite determination.

### Species identification

**Figure 1:**
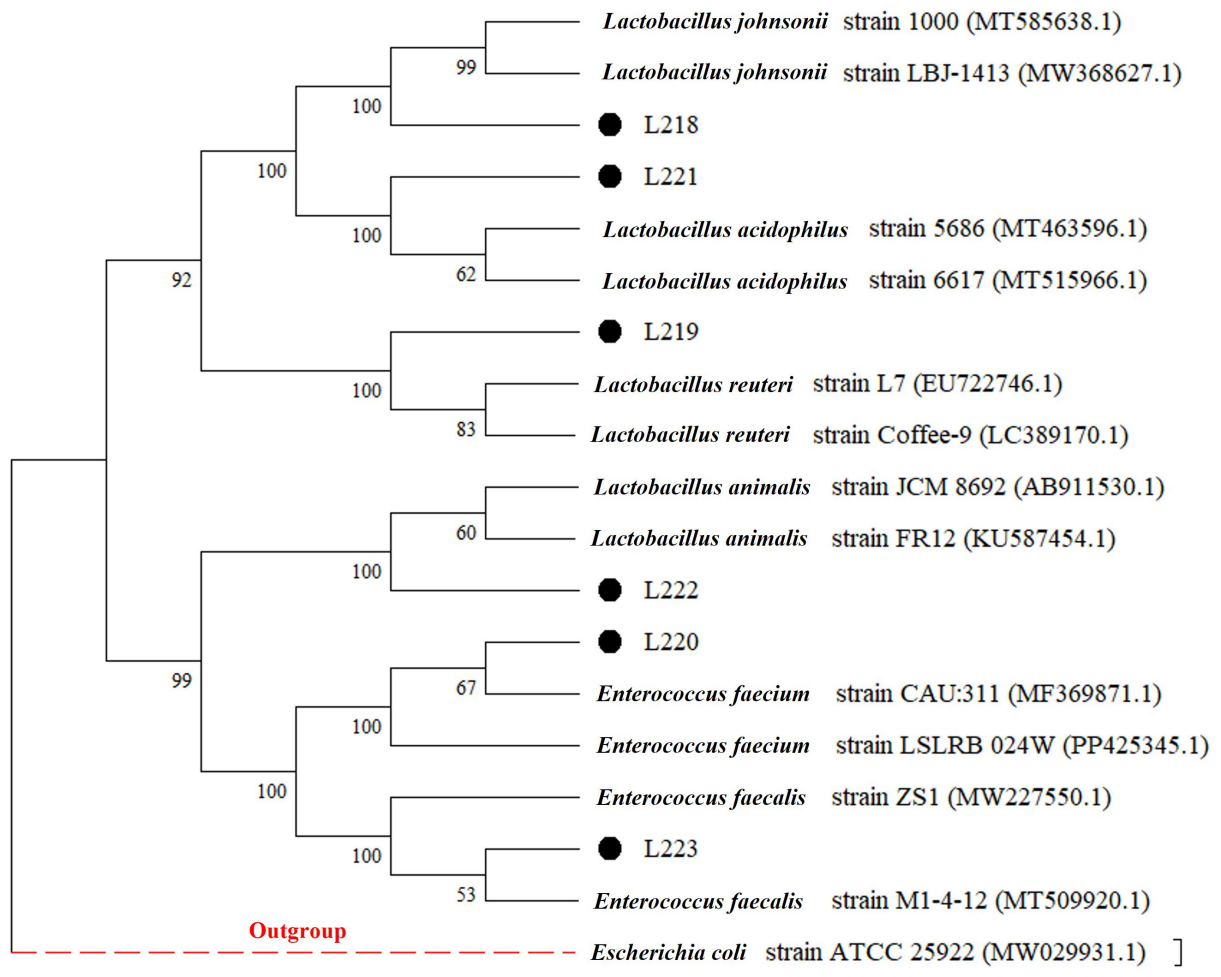
Phylogenetic tree (based on neighbor-joining method) of six LAB strains created with the data from 16S rRNA gene analysis results (Bootstrap value was 1,000 repeats. *Escherichia coli* ATCC 25922 was used as outgroup).

The 16S rRNA sequences of the 6 LAB isolates obtained were compared with the sequences in GenBank. The strains were identified as members of the Lactobacillus and Enterococcus genera, which are commonly recognized as lactic acid bacteria. Specifically, the strains were related to *Lactobacillus johnsonii* (L218), *Lactobacillus reuteri* (L219), *Enterococcus faecium* (L220), *Lactobacillus acidophilus* (L221), *Lactobacillus animalis* (L222), and *Enterococcus faecalis* (L223), with sequence similarity exceeding 98%. The phylogenetic tree, constructed based on the 16S rRNA gene sequences and shown in Figure 1, provides a visual representation of the genetic relatedness among these isolates.

### Growth performance

**Figure 2:**
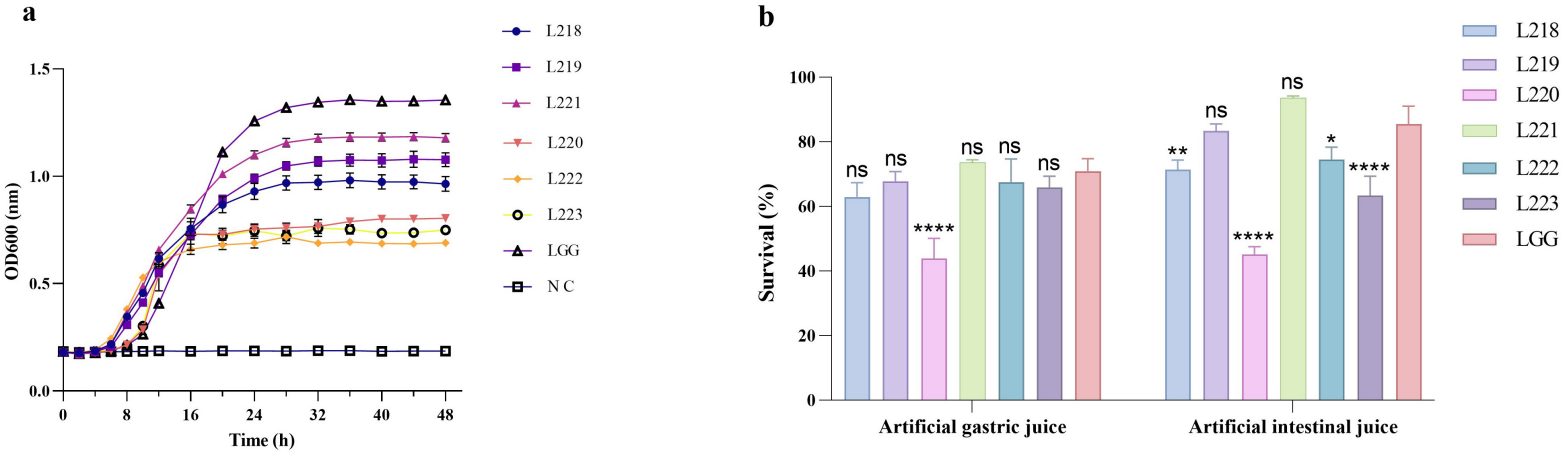
**(a)** Growth curve; **(b)** survival of the potential probiotic isolates in the artificial gastric and intestinal juices. *p < 0.05; **p < 0.01; ***p < 0.001; ****p < 0.0001; ns, not significant (*p* ≥ 0.05). These comparisons are specifically made relative to the control group (LGG).

The growth conditions of the six lactic acid bacteria strains and the control strain LGG are shown in Figure 2a. It was obvious that the growth cycles of all strains were relatively similar, entering the exponential growth phase after about 4 h, and the OD600 value increased exponentially. After 24 h of culture, the growth rate decreased and entered a stable phase. Among them, *Lactobacillus rhamnosus* LGG showed a significantly stronger growth speed and performance compared to the other strains.

### Resistant capacity

Figure 2b shows the survival rate results of LAB strains under simulated gastrointestinal tract (GIT) conditions. After being treated with artificial gastric juice for 3 h, except for Enterococcus faecium L220, other isolated strains showed higher resistance to simulated gastric juice, with survival rates exceeding 60%. The specific average survival rates for these strains ranged from 43.91 to 73.65%, and there was no significant difference compared with the control group, which was treated with the same artificial gastric juice (*P* > 0.05). Seven hours after incubating the surviving cells in artificial intestinal fluid, strains L221 (93.59%) and L219 (83.39%) showed better resistance to artificial intestinal fluid, with no significant difference compared with the control group (*P* > 0.05).

## Antipathogenic activity detection

### Antibacterial activity

Table 1 shows that the six strains exhibit varying levels of antagonistic activity against four common intestinal pathogenic bacteria. The control strain LGG exhibited the strongest antagonistic activity against *Escherichia coli* and *Staphylococcus aureus*. Its antagonistic activity against *Escherichia coli* was significantly better than that of the other isolated strains (*P* < 0.05). L221 and L222 were the isolates with the strongest antagonistic activity against *Salmonella* and *Pseudomonas aeruginosa*, respectively. Both were superior to the control strain LGG (*P* < 0.05).

**Table 1:** Antagonistic activity of potential probiotic strains from canine milk samples against pathogenic bacteria by the Oxford cup method.

**Strain**	**Diameter of inhibition zone (mm)**			
	***E. coli***	***Staphylococcus aureus***	***Salmonella***	***P. aeruginosa***
	**ATCC 25922**	**ATCC 25923**	**H9812**	**PAO 1**
L218	11.07 ± 0.46^d^	15.60 ± 0.17^ab^	16.65 ± 1.21^c^	22.61 ± 0.44^b^
L219	13.36 ± 0.95^c^	16.21 ± 1.27^a^	17.95 ± 0.31^b^	12.95 ± 0.49^f^
L220	11.78+0.22^d^	14.26+0.61^c^	15.81+0.41^cd^	15.11+0.51^e^
L221	13.63 ± 0.50^c^	16.34 ± 0.63^a^	21.32 ± 0.95^a^	18.00 ± 0.67^c^
L222	12.91+0.41^c^	14.56+0.29^bc^	15.18+0.60^d^	23.80+0.58^a^
L223	14.96+0.45^b^	13.67+0.16^c^	16.04+0.17^cd^	18.57+0.29^c^
LGG	21.37+0.07^a^	16.65+0.52^a^	18.52+0.42^b^	16.91+0.35^d^

All the results are represented as mean ± SD.

Different letters indicate significant differences (Waller-Duncan, *p* < 0.05) in the columns.

### Co-aggregative ability with pathogens

Table 2 shows that the co-aggregation rates of the six LAB strains against four common pathogens. The strains with the highest co-aggregation ability against all four pathogens was Lactobacillus reuteri strain L219, including *Escherichia coli* (68.03%), *Staphylococcus aureus* (69.70%), *Salmonella* (68.10%), and *Pseudomonas aeruginosa* (69.60%).

**Table 2:** Co-aggregative activity of potential probiotic strains from canine milk samples against pathogenic bacteria.

**Strain**	**Co-aggregative ratio (%)**			
	***E. coli***	***Staphylococcus aureus***	***Salmonella***	***P. aeruginosa***
	**ATCC 25922**	**ATCC 25923**	**H9812**	**PAO 1**
L218	47.44 ± 1.81^e^	53.35 ± 1.62^d^	52.94 ± 1.18^c^	58.27 ± 1.53^bc^
L219	68.03 ± 0.98^a^	69.70 ± 0.71^a^	69.60 ± 0.74^a^	68.10 ± 0.84^a^
L220	52.89 ± 1.09^d^	44.94 ± 1.22^e^	53.10 ± 0.83^c^	55.69 ± 2.01^c^
L221	59.40 ± 1.17^c^	62.65 ± 0.70^b^	62.05 ± 1.28^b^	62.26 ± 0.14^abc^
L222	63.19 ± 1.78^b^	35.89 ± 0.83^g^	47.34 ± 1.98^d^	65.21 ± 4.40^ab^
L223	49.93 ± 0.43^e^	42.40 ± 0.47^f^	52.79 ± 1.68^c^	64.26 ± 0.15^ab^
LGG	55.32 ± 1.16^d^	57.33 ± 1.05^c^	65.25 ± 1.85^b^	68.81 ± 0.84^a^

All the results are represented as mean ± SD.

Different letters indicate significant differences (Waller-Duncan, *p* < 0.05) in the columns.

### Adhesion activity detection

To evaluate the adhesion ability of the six isolates, we performed the following experiments: auto-aggregation assay, adhesion ability assay of Caco-2 cell line, cell surface hydrophobicity assay, and biofilm formation ability assay.

### Auto-aggregation activity

**Figure 3:**
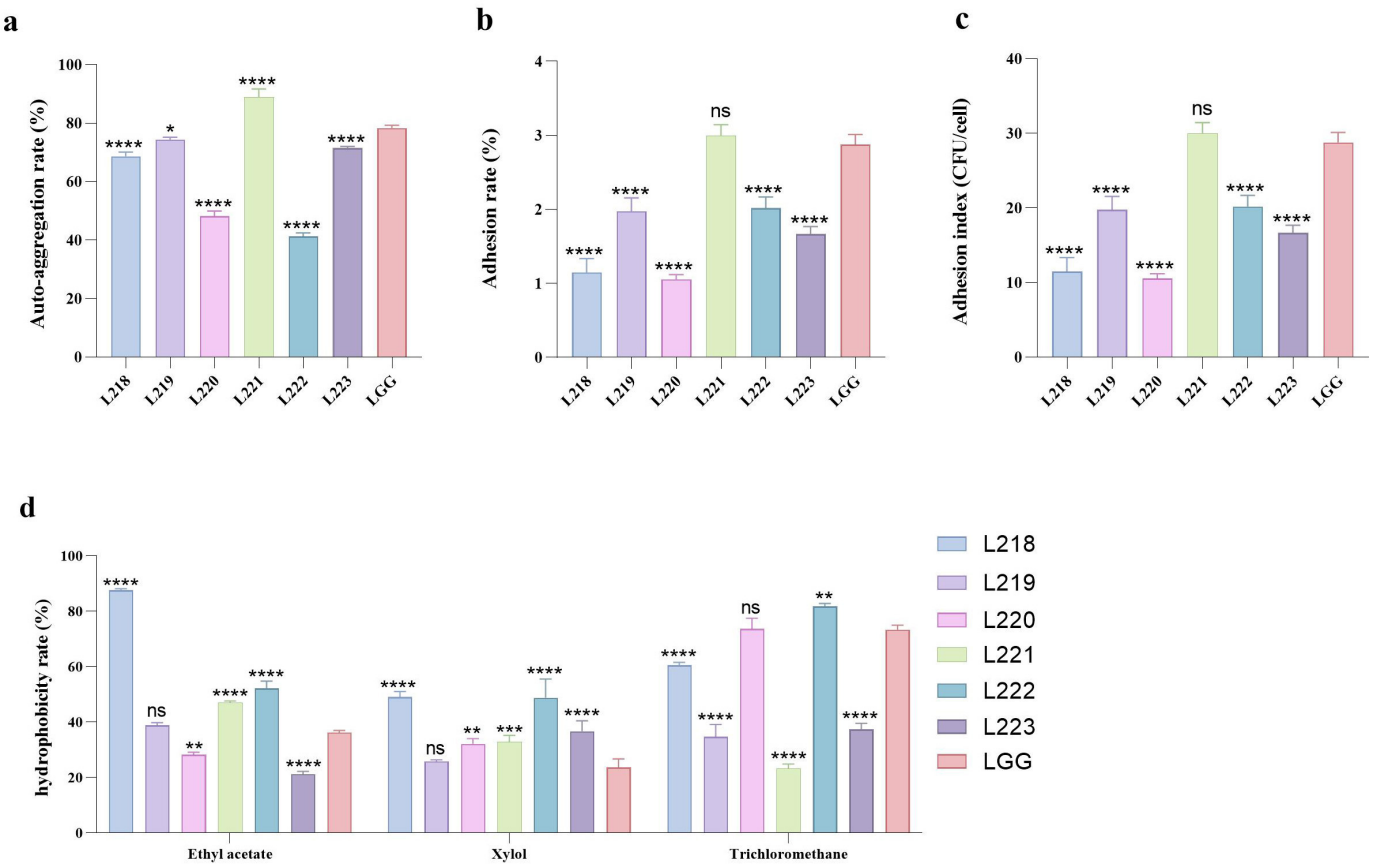
All the results are represented as mean ± SD. **(a)** Auto-aggregation rate of LAB strains. **(b)** Adhesion rate of LAB strains to Caco-2 cell lines. **(c)** Adhesion index of LAB strains to Caco-2 cell lines. **(d)** The cell surface hydrophobicity of LAB strains. *p < 0.05; **p < 0.01; ***p < 0.001; ****p < 0.0001; ns, not significant (*p* ≥ 0.05). These comparisons are specifically made relative to the control group (LGG).

The auto-aggregation ability is shown in Figure 3a. L221 (88.93%) has the highest self-agglutination rate, followed by LGG (78.31%) and L219 (74.40%).

### Adhesion to Caco-2 cells

Figures 3b, c show the adhesion ability of the six LAB strains to the Caco-2 cell line. Among them, Strain L221 showed the strongest adhesion ability (adhesion rate 3.00%, 30.00 CFU/cell), followed by control strain LGG (adhesion rate 2.88%, 28.77 CFU/cell).

### Cell surface hydrophobicity

Figure 3d displays the results of cell surface hydrophobicity. Significant differences were observed in the hydrophobicity of various LAB strains when exposed to different solutions. L218 exhibited the highest hydrophobicity against two organic solvents: Ethyl acetate (87.63%) and Xylol (49.03%). L222 exhibited the highest hydrophobicity toward the organic solvent Trichloromethane (81.76%).

### Determination of biofilm forming ability

Supplementary Table 2 displays the biofilm formation ability of the six isolates. Strains L218, L219, L220, and L223 exhibited moderate biofilm formation ability (++), while strain L221, L222 and LGG demonstrated strong biofilm formation ability (+++).

## Safety assessment

### Hemolytic activity

The hemolytic activity test results showed that these six isolates were non-hemolytic (Supplementary Figure 1).

### Antibiotic susceptibility

Table 3 shows the susceptibility results of six isolates to 14 antibiotics. The resistance rates (includes resistance and intermediate) were 0% (0/6) to penicillin G, 0% (0/6) to amoxicillin, 0% (0/6) to amoxicillin, 16.67% (1/6) to erythromycin, 0% (0/6) to Cefuroxim, 0% (0/6) to cefotaxime, 83.33% (5/6) to Oxacillin, 0% (0/6) to Cefazolin, 100% (6/6) to Norfloxacin, 33.33% (2/6) to Rifampicin, 16.67% (1/6) to clindamycin, 0% (0/6) to chloramphenicol, 33.33% (2/6) to tetracycline, 100% (6/6) to vancomycin. Isolates L219, L221, and L222 had the highest sensitivity to 14 antibiotics (78.57%), followed by L218 (71.43%), L220 (64.29%), and L223 (64.29%).

**Table 3:** Antibiotic susceptibility of 6 LAB strains isolated from canine milk to different antibiotics.

**Strain**	**Antibiotic susceptibility**														**Sensitive rate (S + I, %)**
	**P**	**AMP**	**AML**	**E**	**CXM**	**CTX**	**OX**	**KZ**	**NOR**	**RD**	**DA**	**C**	**TE**	**VA**	
L218	S	S	S	R	S	S	S	S	R	S	R	S	S	I	71.43
L219	S	S	S	S	S	S	R	S	R	S	S	S	S	R	78.57
L220	S	S	S	S	S	S	R	S	R	R	S	S	R	R	64.29
L221	S	S	S	S	S	S	R	S	R	S	S	S	S	R	78.57
L222	S	S	S	S	S	S	R	S	R	S	S	S	S	R	78.57
L223	S	S	S	S	S	S	R	S	R	I	S	S	R	R	64.29

P, penicillin G; AMP, ampicillin, AML, amoxicillin; E, erythromycin; CXM, Cefuroxim; CTX, cefotaxime; OX, Oxacillin; KZ, Cefazolin; NOR, Norfloxacin; RD, Rifampicin; DA, clindamycin; C, chloramphenicol; TE, tetracycline; VA, vancomycin.

R, resistance; S, sensitive; I, intermediate.

## Antioxidant capacity assessment

### Tolerance to H_2_O_2_

Table 4 displays the tolerance of 6 LAB strains to varying concentrations of H_2_O_2_. The results showed that all six isolates could survive well in 0.5 mmol/L H_2_O_2_, with survival rates ranging from 86.01 to 98.96%. Except for isolates L220 and L221, the survival rates of the other strains were found to be statistically significantly higher than the control strain LGG (*P* < 0.05). When the concentration increased to 1 mmol/L H_2_O_2_, the survival rate of strains L220, L221, and LGG exceeded 28%. When the concentration increased to 1.5 and 2 mmol/L H_2_O_2_, the overall survival rate was lower than 10%.

**Table 4:** Livability of potential probiotic strains from canine milk samples in different concentrations of H_2_O_2_ environment.

**Strain**	**Survival rate (%)**			
	**0.5 mmol/L H**_2_ **O**_2_	**1.0 mmol/L H**_2_ **O**_2_	**1.5 mmol/L H**_2_ **O**_2_	**2.0 mmol/L H**_2_ **O**_2_
L218	98.90 ± 4.01^a^	3.01 ± 0.12^e^	1.96 ± 0.08^f^	1.41 ± 0.07^d^
L219	98.96 ± 0.83^a^	3.68 ± 0.01^de^	2.93 ± 0.12^d^	2.40 ± 0.05^c^
L220	86.01 ± 0.89^b^	34.24 ± 0.52^b^	2.89 ± 0.09^d^	1.45 ± 0.36^d^
L221	90.22 ± 4.22^b^	41.88 ± 2.28^a^	10.49 ± 0.28^a^	4.37 ± 0.10^b^
L222	97.59 ± 1.23^a^	5.14 ± 0.23^d^	2.59 ± 0.07^e^	2.21 ± 0.01^c^
L223	94.56 ± 0.63^a^	3.61 ± 0.32d^e^	3.64 ± 0.03^c^	1.39 ± 0.08^d^
LGG	87.42 ± 1.39^b^	28.97 ± 0.43^c^	8.13 ± 0.27^b^	4.69 ± 0.22^a^

All the results are represented as mean ± SD.

Different letters indicate significant differences (Waller-Duncan, *p* < 0.05) in the columns.

### Free radical scavenging ability

The results of the free radical scavenging experiment are shown in Table 5. The cell-free supernatant of the six LAB isolates was always better than the bacterial suspension. The clearance rate of DPPH by bacterial suspension is 18.05% ± 0.15 to 24.40% ± 0.35, all were higher than the control strain LGG (17.65 ± 0.36). The highest clearance rate (24.40 ± 0.35) was observed in strain L219. Interestingly, the overall DPPH clearance rate of the supernatant for the isolated strains remained within the range of 77.91% ± 1.09 to 88.44% ± 0.99, all lower than the control strain LGG (93.43 ± 1.16). The clearance rate of OH^−^ in bacterial suspension is 11.27% ± 1.05 to 22.38% ± 3.73, all lower than the control strain LGG (39.67 ± 1.35). The removal rate of OH- in the supernatant liquid is 58.00–71.40%. Strain L223 showed the highest removal rate (71.40 ± 1.57). The isolated strains showed no ability to clear O2^−^ from bacterial suspensions or cell-free supernatants, which is consistent with the control strain LGG.

**Table 5:** DPPH, OH^−^, and O2^−^ radical scavenging activity of 6 LAB strains isolated from canine milk samples.

**Strain**	**DPPH scavenging rate (%)**		**OH** ^**−**^ **scavenging rate (%)**		**O** ^**2−**^ **scavenging rate (%)**	
	**Supernatant**	**Suspension**	**Supernatant**	**Suspension**	**Supernatant**	**Suspension**
L218	85.07 ± 2.14^c^	19.69 ± 0.06^bc^	62.49 ± 9.22^b^	20.20 ± 2.56^bc^	0.00 ± 0.00	0.00 ± 0.00
L219	87.40 ± 0.96^bc^	24.40 ± 0.35^a^	70.63 ± 4.57^a^	13.45 ± 1.84^e^	0.00 ± 0.00	0.00 ± 0.00
L220	86.51 ± 1.06^bc^	18.68 ± 1.43^cd^	44.25 ± 2.57^d^	17.37 ± 0.82^cd^	0.00 ± 0.00	0.00 ± 0.00
L221	88.44 ± 0.99^b^	20.13 ± 0.17^b^	70.73 ± 4.10^a^	22.38 ± 3.73^b^	0.00 ± 0.00	0.00 ± 0.00
L222	86.18 ± 0.97^bc^	18.05 ± 0.15^d^	58.00 ± 1.76^c^	11.27 ± 1.05^e^	0.00 ± 0.00	0.00 ± 0.00
L223	77.91 ± 1.09^d^	18.58 ± 0.19^d^	71.40 ± 1.57^a^	14.39 ± 1.18^de^	0.00 ± 0.00	0.00 ± 0.00
LGG	93.43 ± 1.16^a^	17.65 ± 0.36^d^	63.43 ± 3.07^b^	39.67 ± 1.35^a^	0.00 ± 0.00	0.00 ± 0.00

All the results are represented as mean ± SD.

Different letters indicate significant differences (Waller-Duncan, *p* < 0.05) in the columns.

## Metabolite determination

**Figure 4:**
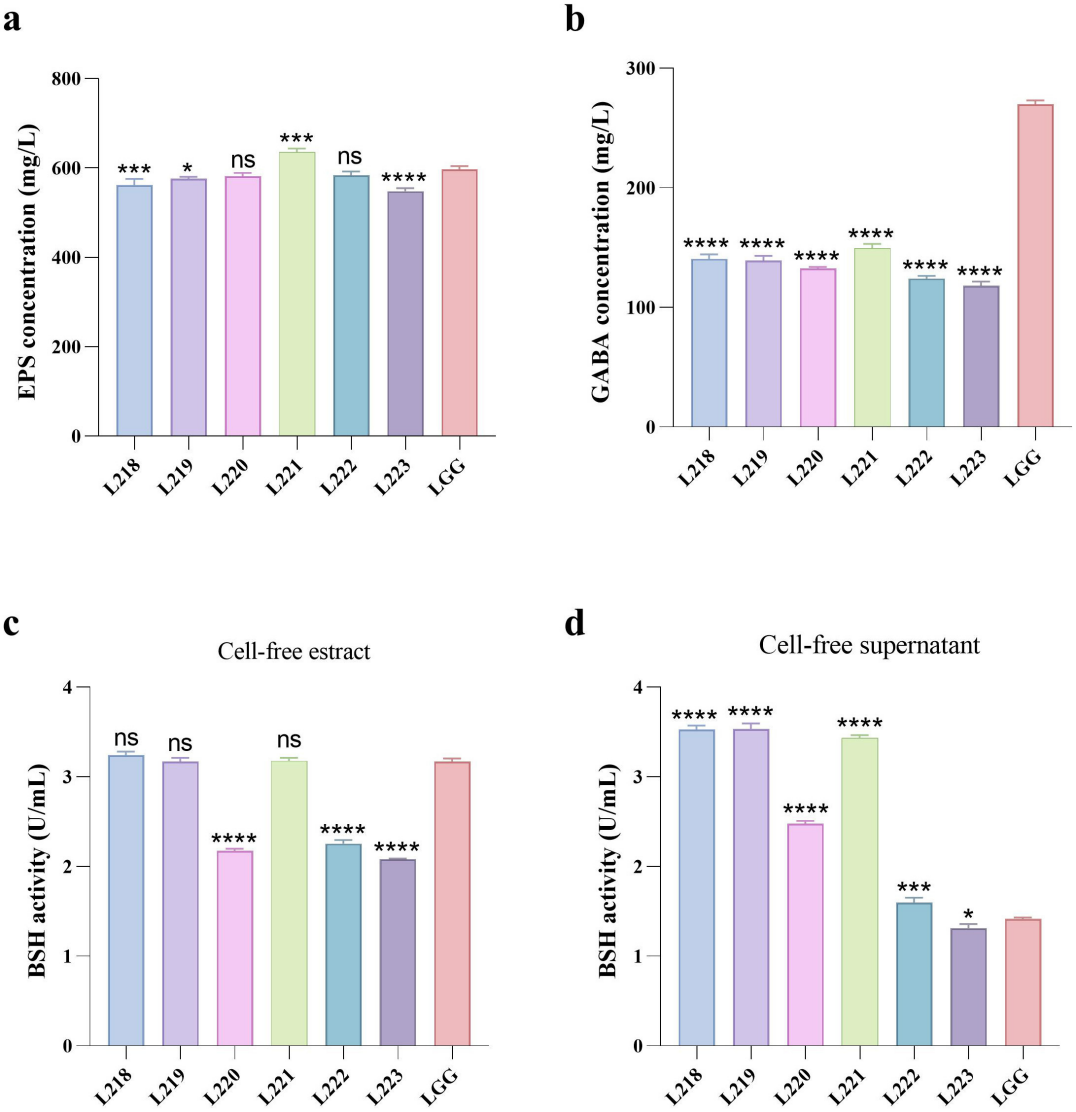
Metabolites production abilities of seven LAB strains. All the results are represented as mean ± SD. **(a)** EPS production ability. **(b)** GABA production ability. **(c)** BSH production ability of cell-free extract. **(d)** BSH production ability of cell-free supernatant. *p < 0.05; **p < 0.01; ***p < 0.001; ****p < 0.0001; ns, not significant (*p* ≥ 0.05). These comparisons are specifically made relative to the control group (LGG).

Figure 4 shows the results of metabolite assessment for six isolates. All strains showed good EPS production capacity, with values exceeding 548.52 mg/L (Figure 3a). Strain L221 has the highest EPS production capacity of 635.81 mg/L, significantly higher than the control strain LGG (597.09 mg/L).

The GABA production capacity of the six isolates ranged from 118.19 to 149.51 mg/L (Figure 3b), Significantly lower than the control strain LGG (269.81 mg/L). The BSH production capacity of cell-free extracts of the isolates ranged from 2.08 to 3.24 U/mL (Figure 3c), while the cell-free supernatants of the isolates ranged from 1.31 to 3.54 U/mL (Figure 3d).

## Discussion

In this study, we isolated six LAB strains from canine milk samples via 16S rRNA molecular identification. The results of the basic characteristics test showed that all six strains exhibited robust growth and demonstrated resistance to the artificial gastrointestinal tract environment. Among them, *Lactobacillus acidophilus* L221 performed best, possibly due to its unique cell wall structure and acid and bile resistance mechanisms (37, 38). The growth curve reflects the growth and metabolic capabilities of bacteria, suggesting their potential for rapid activation upon reaching the intestinal colon and their ability to establish and maintain a large number of colonies for a long time, laying the foundation for their probiotic effects in animal intestines (39). The strains' good tolerance to the gastrointestinal tract environment, as assessed by their ability to survive in artificial gastric and intestinal fluids, suggests that they can reach the intestine intact and undergo further reproductive growth (40).

Anti-pathogenic activity and safety properties are considered the most important properties for probiotics. In dogs, prevalent bacterial pathogenic microorganisms such as *Escherichia coli, Staphylococcus aureus, Salmonella*, and *Pseudomonas aeruginosa* are responsible for a spectrum of diseases including ulcerative keratitis, otitis media, pyoderma, urinary tract infections, skin and wound infections, as well as various respiratory tract infections (41–46). In this study, we found that the cell-free supernatants derived from six isolates exhibited robust antagonistic activity against these four common pathogens. This suggests that our LAB candidate strains hold promise for potential applications in the treatment and prevention of these diseases in dogs in the future.

The functionality of LAB in resisting pathogenic bacterial invasion, maintaining intestinal flora balance, and modulating immune responses is contingent upon their adherence to host intestinal epithelial cells (47). This adhesion is related to cell surface hydrophobicity and autoaggregation activity. Enhanced cell surface hydrophobicity facilitates interactions between LAB strains and intestinal epithelial cells, while auto-aggregation activity enables LAB strains to achieve high cell densities in the intestine (48). In this study, six canine-derived LAB strains were found to be hydrophobic to organic solvents (ethyl acetate, xylene, and chloroform). In addition, these strains showed better self-agglutination ability and adhesion to Caco-2 cells. Notably, *Lactobacillus acidophilus* (L221) exhibited the most robust properties, which may be attributed to the strain's ability to produce a large amount of EPS. Similar results were reported by Kos et al. for *Lactobacillus acidophilus* M92 (26). Compared to probiotics in the free state, probiotics in the periplasmic state have been shown to have excellent gastrointestinal tolerance and adhesion capabilities (49). In this study, all six potential LAB strains showed strong biofilm forming ability, suggesting their capacity to adhere, colonize, and replicate in the gastrointestinal tract.

When the body undergoes oxidative stress, it produces large amounts of reactive oxygen species (ROS), including H_2_O_2_, DPPH, OH^−^, and O^2−^ radicals. Excess ROS attack proteins, lipids, nucleic acids, and other biomolecules, further exacerbating oxidative stress. Oxidative damage to these biomolecules can trigger apoptosis and contributing to various diseases such as inflammation, cancer, atherosclerosis, aging, and degenerative diseases (50). Numerous studies have demonstrated that LAB possess a potent antioxidant capacity, capable of inhibiting oxidative stress and mitigating the damage caused by associated diseases (51). *Lactobacilli* have been shown to exert their antioxidant capacity through both ROS scavenging and redox systems (52–54). It is noteworthy that the antioxidant capacity and mechanisms may vary among different LAB species. In this study, the six canine-derived LAB strains demonstrated high tolerance to 0.5 mmol/L H_2_O_2_, as well as high scavenging capacity for DPPH and OH^−^ radicals. However, they did not show any O^2−^ radical scavenging capacity, which is consistent with the findings of Kuda et al. (55). In addition, *Lactobacillus acidophilus* L221 showed the ability to tolerate 1.0 and 1.5 mmol/L H_2_O_2_ and the strongest scavenging ability against DPPH and OH^−^ radicals. *Lactobacillus acidophilus* L221 exhibited the best comprehensive *in vitro* antioxidant effect, likely due to its production of antioxidative metabolites, such as γ-aminobutyric acid (GABA) and exopolysaccharides, which enhance its ability to scavenge ROS (56).

Beneficial metabolite production is a crucial factor in evaluating functional probiotics. EPS, produced by *Lactobacillus* during reproduction and metabolism, is an important metabolite that promotes animal health. Studies have shown that EPS can have beneficial effects on the organism through antibacterial, antiviral, antioxidant, antitumor, and immunomodulatory effects (9). Therefore, the screening for LAB strains that produce EPS and the quantitative analysis of EPS have garnered considerable attention. Hamet et al. screened 28 strains of *Lactobacillus* spp with EPS production capacities ranging from 20 to 370 mg/L (57). However, the six strains of LAB strains evaluated in this study demonstrated a strong EPS production capacity ranging from 548.52–635.81 mg/L. The highest strain was *Lactobacillus acidophilus* L221, suggesting potential multifunctional effects. This study is one of the few to evaluate the EPS production capacity of LAB canis. LAB produces GABA, an inhibitory neurotransmitter in the mammalian central nervous system. GABA has been investigated for its physiological roles, such as stimulating appetite, aiding digestion, managing epilepsy, suppressing cancer cell growth, and boosting immune function (58, 59). Therefore, the screening of GABA-producing LAB is a current research focus. However, there are few reports on GABA-producing LAB of canine origin. In this experiment, we discovered that 6 strains of LAB strains produced 118.18–149.51 g/L of GABA, which is comparable to the 0.16 g/L produced by *Lactobacillus plantarum* 8014 as reported by Li et al. (60). This suggests that the six LAB strains have the potential to be probiotics. Bile salt hydrolase (BSH) is an intracellular enzyme produced by intestinal flora during growth and reproduction. It regulates the balance of bile acids in the host, affects lipid metabolism, and controls cholesterol, as well as regulates intestinal diseases (61). Therefore, it is important to screen for LAB that produce BSH. Pinto et al. found that BSH activity was absent in all seven Lactobacillus isolates examined (62). Tsai et al. screened 800 strains of Lactobacillus and found only 22 with BSH activity (63). In the present study, six strains of LAB were found to have BSH activity, ranged from 1.31 to 3.54 u/ml, suggesting potential probiotic functions.

Compared to other well-known probiotic strains, L221 demonstrated a distinct advantage, particularly in its strong gastrointestinal tolerance, superior adhesion ability, robust anti-pathogenic activity against common bacterial pathogens, and potent antioxidant effects (8, 27, 64). These exceptional attributes not only highlight L221's potential in enhancing gut health and modulating immune responses, but also position it as a versatile probiotic with applications in the treatment and prevention of various canine diseases (10). Given the similarities in gastrointestinal microbiota across species, the strain's ability to improve gut health and support immune function may extend beyond dogs, offering potential benefits for other companion animals, livestock, and even human health applications. However, some limitations should be acknowledged, such as the lack of *in vivo* validation of L221's efficacy and safety in dogs, as well as the need for long-term studies to assess its potential side effects. Future research could focus on evaluating the long-term therapeutic effects of L221 in canine health and exploring its interactions with other gut microbiota.

## Data Availability

The original contributions presented in the study are included in the article/Supplementary material, further inquiries can be directed to the corresponding author.
