# Supplementary material for: Isolation and evaluation of multi-functional properties of lactic acid bacteria strains derived from canine milk
Source: Front Vet Sci. 2024 Nov 28;11:1505854. doi: 10.3389/fvets.2024.1505854 (PMC11634844; doi:10.3389/fvets.2024.1505854)
Supplement: Supplementary file 1 [file Supplementary_file_1.docx]

Supplementary Material

# Supplementary Figures and Tables

## Supplementary Table

Supplementary Table 1 Characterization of LAB strains isolated from canine milk samples based on Gram staining, cell morphology, and catalase.

| LAB strains | Gram Staining | Morphology | Catalase |
| --- | --- | --- | --- |
| L218 | + | Rod | − |
| L219 | + | Rod | − |
| L220 | + | Cocci | − |
| L221 | + | Rod | − |
| L222 | + | Rod | − |
| L223 | + | Cocci | − |

Note: +, positive; −, negative.

Supplementary Table 2 Biofilm formation capacity of 7 LAB strains isolated from canine milk.

| Strain | Biofilm formation capacity |
| --- | --- |
| L218 | ++ |
| L219 | ++ |
| L220 | ++ |
| L221 | +++ |
| L222 | +++ |
| L223 | ++ |
| LGG | +++ |

## Supplementary Figures


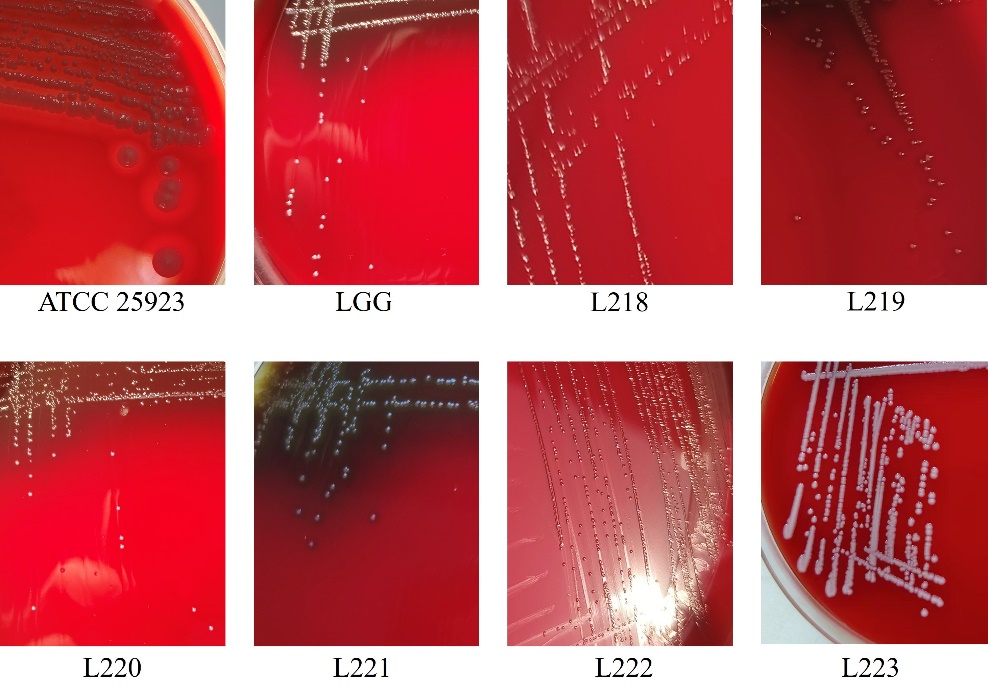


Supplementary Figure 1 The results of the hemolytic test of LAB strains and *Staphylococcus aureus* ATCC 25923.
